# Foot Function Index for Arabic-speaking patients (FFI-Ar): translation, cross-cultural adaptation and validation study

**DOI:** 10.1186/s13018-022-03092-7

**Published:** 2022-04-07

**Authors:** Shershah Khan, Suzanne Faulkner, Fahad S. Algarni, Abdulaziz Almalki, Ahmed Almansour, Abdulrahman M. Altowaijri

**Affiliations:** 1 Medical Rehabilitation Department, King Saud University Medical City, King Saud University, Riyadh, 11461 Saudi Arabia; 2 National Centre for Prosthetics and Orthotics, Department of Biomedical Engineering, Wolfson Building, University of Strathclyde, Glasgow, UK; 3 Department of Rehabilitation Sciences, College of Applied Medical Sciences, King Saud University, Riyadh, 11433 Saudi Arabia; 4 Department of Physical Therapy, College of Applied Medical Science, Majmaah University, Al Majma’ah, Saudi Arabia

**Keywords:** Foot Function Index, FFI, Outcome assessment, Translation and validation

## Abstract

**Background:**

Foot Function Index (FFI) is a valid and reliable outcome measure, which is widely used to measure the foot and ankle functional level and disorders. Until now, no validated Arabic version of the FFI is available. This study was conducted at a tertiary care hospital in Riyadh, Saudi Arabia. The purpose of this project was to translate and adapt the FFI into Arabic and to evaluate its psychometric properties of validity and reliability.

**Methods:**

The study consisted of two phases. The first phase was the translation and cultural adaptation of the FFI to Arabic. The next phase involved, testing the psychometric properties of the Arabic version of the FFI on a sample of 50 consecutive participants which included internal consistency, test–retest reliability, floor and ceiling effects and construct validity.

**Results:**

The mean age of the study participants was 38 ± 12.94 years. Both the genders were evenly enrolled with 50% of the participants as male and 50% as female. Majority of them complained of plantar fasciopathy (32%) followed by pes planus (22%) and ankle sprain (18%). The scores of FFI-Ar were normally distributed, confirmed by a significant Shapiro–Wilk test. The mean value of FFI-Ar total score was 47.73 ± 19.85. There were no floor or ceiling effects seen in any of the subscales and total score. The internal consistency was good with the Cronbach’s alpha value of 0.882, 0.936 and 0.850 for the pain, disability and activity limitation subscales, respectively. The reproducibility of the FFI-Ar was analysed by intra-class correlation coefficient which revealed good to excellent test–retest reliability. A significant correlation was found between FFI-Ar and SF-36 and numeric rating scale (NRS) confirming its construct validity.

**Conclusion:**

The FFI-Arabic version showed good validity and reliability in patients with foot and ankle problems. This tool can be used in usual practice and research for analysing foot and ankle disorders in Arabic-speaking people.

**Supplementary Information:**

The online version contains supplementary material available at 10.1186/s13018-022-03092-7.

## Introduction

Foot and ankle pathologies are often secondary to traumatic and non-traumatic problems, and include but not limited to: plantar fasciopathy, hallux valgus, metatarsalgia, hammer toe, ankle sprain, osteoarthritis and rheumatoid arthritic changes [1, 2]. The prevalence of foot problems ranges from 6 to 30% in general population [3, 4]. The situation becomes more apparent in the aging population. Persistent painful foot conditions occur in about 24% of older adults [5]. Individuals aged 65 and above are generally considered as older adults [6]. Such conditions have been treated by different approaches. Patient-reported outcome measures (PROMs) are very helpful tools in assessing the efficacy of the response to these treatments [7]. Currently, there are several PROMs used in foot and ankle research.

The Foot Function Index (FFI) is a self-reporting outcome measure developed by Budiman-Mak et al. [8]. In a systematic review by Rosenbaum et al., the FFI was identified as the most widely used foot complaint-related evaluation tool [9]. This instrument is found to be feasible, easily calculated, easily understood by the participants and takes less than 10 min to complete [10, 11]. The FFI has been applied to more than 5000 participants worldwide, with 20 different foot and ankle pathologies [11]. The use of FFI is wide and has been employed in studies with orthotic intervention, physical therapy and surgery for various problems [12–15]. It has shown excellent validity, reliability and responsiveness in previous studies [11]. It is also compatible with other outcome measures like SF-36 for analyzing foot and ankle problems [16]. Although the FFI has been translated to numerous languages [10, 16–25] yet no Arabic version has been published. An Arabic version for the general population of Saudi Arabia and Arabic-speaking countries would make it more convenient by providing a resourceful OM tool for evaluating and managing foot and ankle disorders.

Hence, the present study was aimed to translate the FFI into Arabic and adapt it to the local culture. Additionally, the psychometric properties of the Foot Function Index—Arabic (FFI-Ar) version were tested, including the internal consistency, test–retest reliability and construct validity.

## Materials and methods

### Study design

This study was conducted in two phases: i) The translation and cross-cultural adaptation of the original FFI into Arabic (FFI-Ar); ii) the evaluation of the validity and reliability of the FFI-Ar using a cross-sectional study design on a sample of patients having foot and ankle disorders. The International Society for Pharmacoeconomics and Outcomes Research (ISPOR) guidelines were used to perform the adaptation process [26]. This study obtained ethical approval from the University Ethics Committee of University of Strathclyde, Glasgow, UK, and locally from the Institutional Review Board of the College of Medicine, King Saud University, Riyadh, KSA (Research Project No. E-21-5703).

#### Phase 1: translation and cross-cultural adaptation of FFI

The FFI was translated from English into Arabic and culturally adapted using the ISPOR guidelines, which were approved by regulatory agencies such as the Food and Drug Administration and the European Medicines Agency. Translation and cultural adaptation of the FFI into Arabic was not merely a translation process, but one that took into account the cultural, idiomatic, linguistic and contextual aspects related to the translation. In the current study the recent trend of translation and cultural adaption was adapted which has been followed in several other studies [27–29].

In the first step (the forward translation), two independent Arabic translations of the original FFI were produced, by two bilingual translators. They were native Arabic-speaking health professionals, having experience in validation studies. In the following step, both the translated versions were merged together to produce a single reconciled version of FFI-Ar. In the third step, which is the investigation of the standard of the translated version, the FFI-Arabic was moved back into the source language. An independent backward translation of the reconciled FFI Arabic into English was produced by a backward translator. The translator was kept blinded of the original FFI. The next step which was the back translation review, the back-translated FFI and the original versions of FFI were compared by a native English-speaking investigator to investigate any short comings.

After translation and back translation were completed, the Arabic version of the FFI was formatted into the approved layout, which matched the original FFI. The FFI-Ar was applied to 15 patients for pilot testing or cognitive debriefing.

All participants were provided with the pre-final Arabic version of the questionnaire. The mean time to complete the questionnaire was 5.03 min (SD ± 0.76). Most participants did not experience any difficulty completing the questionnaire. However, one participant suggested elaborating the questions in items 13 and 14 by adding the word “during”. Two participants asked for an explanation of the term “assistive devices” in items 22 and 23, and stated that “assistive devices” should be better explained. All comments and suggestions stated by piloting patients were critically reviewed by the project team, and decisions about necessary revisions were made. The final translated version was prepared carefully before being sent for psychometric evaluation.

#### Phase 2: testing the psychometric properties of the FFI-Ar

The internal consistency, test–retest reliability and construct validity of the final version of the FFI-Ar were assessed among patients experiencing foot and/or ankle problems. Construct validity was examined with reference to the SF-36. All patients were living in Riyadh city of Saudi Arabia. Male and female participants between 18 and 70 years, primarily diagnosed with foot and/or ankle disorders and referred to medical rehabilitation department at King Saud University Medical City were invited to participate in this study. The final FFI-Ar was given to 50 native Arabic participants who can read and speak Arabic. Those individuals who were unable to read and speak Arabic and having comorbidities and cognitive problems were excluded from the study. Demographic data concerning each participant were collected using a separate form, while their level of pain was measured using the Numeric Rating Scale (NRS). Each participant completed both the FFI-Ar and the previously validated Arabic version of the SF-36. After a week, thirty percent of the participants with stable symptoms completed the FFI-Ar in order to assess the test–retest reliability of the questionnaire.

### Data Collection

A signed consent form was obtained from each participant before administering the questionnaires. At baseline, all questionnaires were completed by each participant, which included the demographic data sheet, the FFI-Ar and the SF-36. The average time to complete all questionnaires was 10 to 15 min. For test–retest reliability, a sample (n = 16) completed the FFI-Ar for a second time. At the second visit, each participant of the subgroup completed the Global Rating of Change Scale (GRC) before the treatment session to identify participants with stable symptoms who would be involved in retest reliability assessment. Following this, a copy of the FFI-Ar was administered. The second assessment was taken after one to two weeks in order to avoid memorisation and prevent any bias.

### Instruments

The FFI is a self-reporting, region-specific outcome measure. It covers several dimensions of foot function and has been used in relation to several pathologies of the foot and ankle. The FFI-Ar contains 23 items categorized into three subscales: pain, disability and activity limitation. The pain and disability subscales contain 9 items each, while the activity limitation subscale contains 5 items. Previous studies showed that the FFI has high validity, reliability and responsiveness. All participants completed the FFI-Ar and all those who were invited for retesting completed it a second time.

The NRS is a simple, unidimensional measure of pain intensity in adults that is comprised of an 11-point scale [30]. The Short Form 36 Health Survey (SF-36) is a multi-function, general health outcome measure with 36 questions. It produces an 8-scale profile of scores as well as two summary scores (physical and mental measures). The Arabic version has been found to be reliable and equivalent to the original version (English) [31]. The SF-36 – Arabic consists of 36 items that assess the physical and mental components of health according to 8 subscales: Physical functioning (PF), Role limitation due to physical health problems (RP), Bodily pain (BP), General health perceptions (GH), Vitality, energy and fatigue (VT), Social functioning (SF) and Role limitation due to emotional problems (RE).

### Statistical analysis

Data analysis was completed using Statistical Package for the Social Sciences (SPSS) software (IBM SPSS Statistics for Windows, Version 23.0; IBM Corp., Armonk, NY, USA). Descriptive statistics (frequencies (%), means ± standard deviations) for the basic features of the participants’ demographic data were analysed. The Cronbach’s alpha was used to examine the internal consistency of the measurement. The test–retest reliability of the FFI-Ar was assessed using intraclass correlation coefficient (ICC) with a 95% confidence interval (95% CI). The Pearson’s correlation analysis was used to evaluate the construct validity and determine the association between the FFI-Ar and the SF-36 Arabic version and NRS. The p-value of significance was set at *p* ≤ 0.05. In this study, it was hypothesized that the FFI-Ar would reveal moderate to higher correlation with the NRS and physical-related SF-36 domains, i.e. convergent validity, while it would reveal weaker correlation with the mental-related domains of SF-36, i.e. discriminant validity.

## Results

### Demographic characteristics and descriptive statistics

The demographic and clinical data of the 50 participants are presented in Table 1.

**Table 1:** Demographic and clinical data (n = 50)

Variable	Mean ± SD Frequency (%)
Age (years)	38.0 ± 12.9
Gender	
Male	25 (50.0)
Female	25 (50.0)
Weight (kg)	79.8 ± 20.9
Height (cm)	165.9 ± 8.7
BMI	28.9 ± 6.8
Marital status	
Single	19 (38.0)
Married	31 (62.0)
Onset of pain (months)	13.0 ± 12.9
NRS pain	5.3 ± 2.1
Foot/ankle complaint	
ANS	1 (2.0)
Achilles tendinitis	2 (4.0)
Ankle ankyloses	1 (2.0)
Ankle sprain	9 (18.0)
Hallux valgus	4 (8.0)
Metatarsalgia	3 (6.0)
Pes planus	11 (22.0)
Plantar fasciopathy	16 (32.0)
RA of foot/ankle	2 (4.0)
TA injury	1 (2.0)

*SD* standard deviation, *ANS* Accessory Navicular Syndrome, *RA* rheumatoid arthritis, *TA injury*: Achilles tendon injury, *BMI* body mass index, *NRS* numeric rating scale

### FFI-Ar score characteristics

The scores of the FFI-Ar were normally distributed. Descriptive statistics of the FFI-Ar subscales and total scores are listed in Table 2.

**Table 2:** FFI-Ar subscales and total score descriptive statistics (n = 50)

Descriptive statistics	Pain	Disability	Activity limitation	Total
Mean	53.09	43.96	34.13	43.73
Median	58.00	48.50	26.00	42.48
Standard deviation	21.29	25.31	25.86	19.85
Variance	453.27	640.69	668.69	394.15
Skewness	− 0.62	0.02	0.54	− 0.05
Kurtosis	− 0.001	− 1.16	− 0.91	− 1.11
Range	95.00	90.00	89.00	74.83
Minimum	5.00	0.00	0.00	3.83
Maximum	100.00	90.00	89.00	78.67

Time of completion of FFI-Ar	5.72 ± 1.02 min		
Normality test	Statistic	df	Sig
Shapiro–Wilk (FFI-Ar total)	0.963	50	0.123

### Floor and Ceiling effects

No floor or ceiling effects were found in the subscales and the total scores. Table 3 shows the floor and ceiling effects for all the subscales and total score.

**Table 3:** Floor and ceiling scores of the FFI-Ar subscales and total score

Subscale	Floor score	Ceiling score
Pain	2 (4%)	1 (2%)
Disability	1 (2%)	1 (2%)
Activity limitation	4 (8%)	1 (2%)
FFI-Ar Total	1 (2%)	1 (2%)

### Internal consistency

Of the three subscales, internal consistency for the disability subscale was the greatest (Cronbach’s coefficient alpha (α) value 0.94). The Cronbach’s coefficient values were also good for the pain and activity limitation subscales (alpha value of 0.88 and 0.85, respectively). The internal consistency for the FFI-Ar total score was fair (alpha value 0.76). The corrected item-to-total correlations ranged from 0.48 to 0.82. Item 7 (Pain walking with orthotics) showed lowest correlation, while item 5 (Pain walking with shoes) showed the highest correlation. Deleting an item from the FFI-Ar did not significantly change the alpha level; the values ranged from 0.80 to 0.92 when an item was deleted at baseline (Table 4).

**Table 4:** Statistics of the FFI-Ar items

Subscale	Item no	Scale mean if item deleted	± Standard deviation	Corrected item-total correlation	Cronbach's alpha if item deleted
Pain	1	31.92	14.562	0.550	0.876
	2	33.26	14.118	0.643	0.869
	3	32.66	14.052	0.696	0.864
	4	32.94	14.26	0.65	0.87
	5	33.10	13.99	0.82	0.85
	6	33.56	14.08	0.77	0.86
	7	36.36	14.68	0.48	0.88
	8	36.40	14.60	0.52	0.88
	9	32.84	14.18	0.57	0.88
Disability	10	32.64	18.21	0.78	0.92
	11	31.46	17.97	0.81	0.93
	12	30.36	18.08	0.79	0.93
	13	31.34	18.01	0.80	0.93
	14	31.72	18.36	0.70	0.93
	15	31.46	17.81	0.76	0.93
	16	32.52	18.42	0.68	0.93
	17	32.44	18.04	0.82	0.93
	18	30.22	18.12	0.70	0.93
Activity Limitation	19	8.78	8.86	0.71	0.81
	20	9.92	9.61	0.54	0.85
	21	8.06	9.27	0.62	0.83
	22	10.42	8.66	0.72	0.80
	23	10.34	8.56	0.72	0.80

### Test–retest reliability

A test–retest reliability analysis was undertaken on 16 stable participants. Stable participants were those whose conditions had neither improved nor deteriorated significantly between the test and retest interval. Test–retest reliability was tested with the intraclass correlation coefficient (ICC2,1) two-way random-effect model, for participants who completed the FFI-Ar questionnaire two times. The ICC value for pain, disability and activity limitation subscales was 0.81, 0.93 and 0.80, respectively, indicating good to excellent agreement of test–retest reliability. The ICC level for the FFI-Ar total score was also good, with a value of 0.89.

**Fig. 1:**
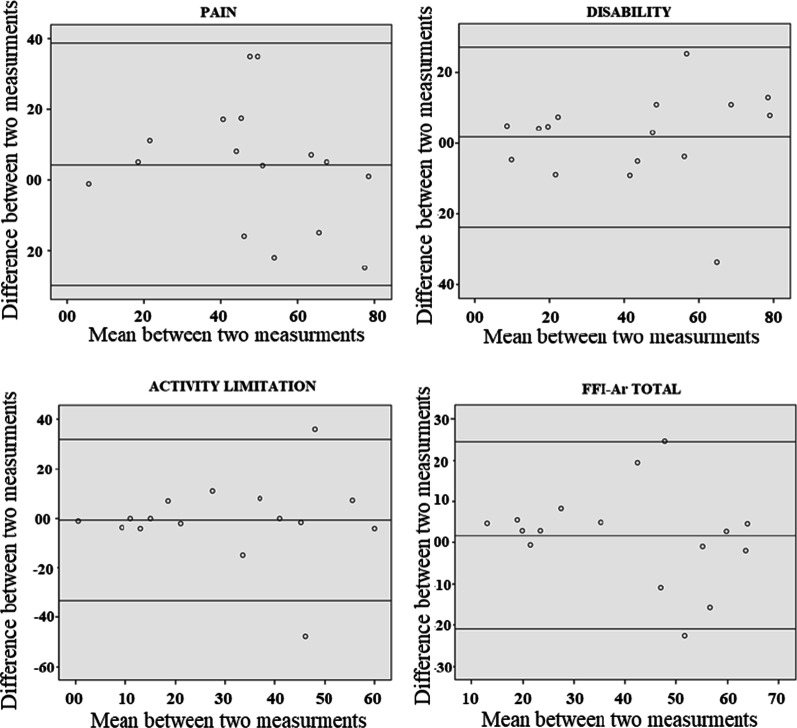
Bland–Altman plot for individual subscales and the total score of FFI-Ar

The test–retest reliability of FFI-Ar statistics is mentioned in Table 5**.** Bland–Altman plots show slight relevant differences from test to retest in the activity limitation subscale, while no significant difference was seen in pain, disability and the FFI-Ar total score (Fig. 1).

**Table 5:** Test–retest reliability of the FFI-Ar

FFI-Ar subscales	Mean (SD)		ICC (95%CI)
	Test score	Retest score	
Pain	50.57 (19.90)	46.41 (24.28)	0.81 (0.48–0.93)
Disability	43.60 (25.32)	41.82 (24.00)	0.93 (0.80–0.97)
Activity limitation	29.83 (19.98)	30.43 (19.96)	0.80 (0.42–0.93)
FFI total	41.33 (17.15)	39.55 (19.84)	0.89 (0.70–0.96)

*ICC* intraclass correlation coefficient, *CI* confidence interval, *95%CI* (lower bound–upper bound)

### Construct Validity

The Pearson’s correlation coefficient (rho) between FFI-Ar and SF-36 resulted in negative values. This explained by the fact that while a higher FFI score indicates worse health status, a higher SF-36 score indicates better health status. Of the physical component of the SF-36, PF, RP and BP had a moderate correlation with all subscales of FFI, while GH showed a weak correlation (Table 6). Alternatively, all four mental component domains of SF-36 (VT, SF, RE and MH) showed weak correlations with the subscales of FFI. The physical component summary (PCS) had a moderate correlation of -0.58 with the FFI total score, while the correlation with the mental component summary (MCS) was negligible. The strongest correlation was between the PCS of SF-36 and disability subscale of FFI (-0.60). Pearson’s correlation coefficients between FFI and SF-36 are described in Table 6.

**Table 6:** Pearson’s correlation coefficient of the FFI subscales with the SF-36 domains

SF-36 domains	Pain	Disability	Activity limitation	FFI total
Physical functioning	− 0.29*	− 0.66**	− 0.61**	− 0.65**
Role physical	− 0.34*	− 0.49**	− 0.43**	− 0.52**
Bodily pain	− 0.58**	− 0.51**	− 0.44**	− 0.61**
General health	− 0.23	− 0.27	− 0.14	− 0.26
Vitality	− 0.30*	− 0.42**	− 0.28	− 0.40**
Social functioning	− 0.35*	− 0.34*	− 0.36**	− 0.43**
Role emotional	− 0.22	− 0.27	− 0.32*	− 0.33*
Mental health	− 0.29	− 0.16	− 0.25	− 0.28*
PCS	− 0.34*	− 0.60**	− 0.47**	− 0.58**
MCS	− 0.26	− 0.19	− 0.25	− 0.28
NRS	0.72**	0.48**	0.24	0.57**

*PCS* physical component summary, *MCS* mental component summary, *NRS* numeric rating scale

**Correlation is significant at the 0.01 level (2-tailed)

*Correlation is significant at the 0.05 level (2-tailed)

Construct validity of FFI-Ar was also confirmed via its strong Pearson’s correlation with NRS pain. The strongest correlation was between the pain subscale of the FFI-Ar and the NRS (rho value of 0.72), while the activity limitation subscale of the FFI-Ar showed a weak correlation. The FFI-Ar total score correlation with the NRS was also good (rho value of 0.57, Table 6)**.** The correlation coefficient between NRS and FFI-Ar resulted in positive values, as higher scores on both scales mean worse health status.

The analysis of correlation upheld the hypothesis of convergent and discriminant validity for FFI-Ar. It was hypothesized that the FFI-Ar would result in a high correlation with the physical domains of SF-36 and NRS and would result in a weak correlation with the mental domains of SF-36. The strong Pearson’s correlation of FFI-Ar with the NRS, physical domains and PCS of SF-36 confirmed its convergent validity. Its weak correlation with the mental domains and MCS of SF-36 proved its discriminant validity.

## Discussion

This study aimed to translate the FFI into Arabic and test the psychometric properties. This study suggests that the FFI-Ar is a reliable and valid tool for assessing foot and ankle problems in Arabic populations.

Cultural adaptation of a questionnaire is very important, as it may lead to systematic errors if not addressed [27]. All discrepancies and issues encountered during the process were cross-checked, proofread and carefully solved. The only cultural adaption necessary occurred during the first phase and involved the term “four blocks” in the twelfth item of the questionnaire. Similar adaption changes were also made in the French and Persian versions [22, 24]. The word “block” is informally used as a unit of distance in North America, which is unfamiliar in Saudi Arabia. It was deduced that one block on average is equal to 150 m. Thus, the term “four blocks” was translated as “600 m” in the Arabic version. The majority of the recruited participants found that the adapted version of the FFI-Ar was simple, clear and understandable with very few issues.

For this study, participants with both acute and chronic painful ankle/foot conditions were enrolled to ensure a wide variety of patient problems were represented. Enrolling multiple foot conditions such as plantar fasciopathy, metatarsalgia, ankle sprain, hallux valgus, hallux rigidus, cavus foot and painful flat feet was also adopted in the Brazilian Portuguese [21], Italian [20] and Chinese [21, 23] validation studies of FFI. Statistical analyses revealed that the Arabic version of FFI was reliable and valid, and could be used with Arabic populations. The mean completion time for the FFI-Ar was 5.7 min, which suggests that the questionnaire is feasible to administer within a clinical setting. Comparably, Budiman et al. reported that the completion time for the original FFI was 5–10 min, while the French version took up to 10 min [24].

Good internal consistency of FFI-Ar was found for both the total score and the three subscales, indicating that all items contribute to measuring the same construct. Among all subscales, the disability subscale was the most internally consistent with the Cronbach’s alpha value of 0.94, followed by the pain (α = 0.88) and activity limitations (α = 0.85) subscales. The internal consistency results revealed that the Cronbach’s alpha values of FFI-Ar were comparable to those obtained in other FFI translations, such as the Turkish version (Cronbach’s α 0.82–0.94) [25] and French version (Cronbach’s α 0.85–0.97) [24]. Unlike other validation studies in which the internal consistency of the activity limitation subscale was much lower [18, 19], the result of the present study was good (alpha value of 0.85), which indicates that this subscale of FFI-Ar is measuring the same construct. As such, the subscale was not removed from the index.

The test–retest reliability of FFI revealed good to excellent results for all subscales and the total score. The test–retest reliability in the disability subscale was excellent (ICC = 0.93). This may be the case because disability is usually a stable symptom that does not fluctuate as commonly as with pain. The ICC values for pain, activity limitation and total score were 0.81, 0.80 and 0.89, respectively. The original FFI version showed ICC values of 0.69, 0.84 and 0.81 for pain, disability and activity limitation, respectively [8]. The test–retest reliability of the FFI-Ar was also comparable to the other versions of FFI [19, 24, 32].

The SF-36 is considered a gold standard for measuring criterion validity of PROMs. This is evident in several validation studies of FFI, where the SF-36 was employed to test their validity [7, 16]. The construct validity of FFI-Ar was confirmed by its moderate to high correlation with SF-36. In the current study, as also observed in the Turkish [25] and German [16] validation studies, the physical domains of SF-36 showed high correlations with the FFI. In this study, Pearson’s correlation coefficient of FFI-Ar total score with the PCS of SF-36 was − 0.578, while its correlation with the MCS was − 0.282. Similarly, in the Turkish version, the FFI total score correlation coefficient with the PCS and MCS of SF-36 was − 0.278 and − 0.127, respectively [25].

The NRS also showed significant correlations with the FFI. The highest correlation was seen between the pain subscale of FFI and NRS (Pearson’s correlation coefficient 0.721). The reason for this high correlation may be that that both scales directly measure pain.

From the correlation analysis, the predefined hypothesis of convergent and discriminant validity was also confirmed. The high correlation coefficients of FFI-Ar with the NRS and PCS of SF-36 prove its convergent validity. The weak correlation coefficients between FFI-Ar, the mental health domains and the MCS of SF-36 prove its discriminant validity.

The rationale for the weak correlation coefficient with the mental health domains of SF-36 is that the FFI does not have mental and psychosocial subscales. Similarly, the three existing subscales have no such items related to mental health or social interaction. Due to a lack of related items, the FFI was criticised by some researchers. For this reason, it was revised in 2006 to produce (FFI-R) by adding more items and a subscale about quality of life and psychosocial activities [7]. However, most of the translation and validation studies were performed on the original version of FFI, which was developed in 1991.

Initially, the FFI was developed merely for rheumatoid arthritis of foot and ankle problems. In more recent years, its use has expanded. It is now utilised for multiple foot problems, including post-surgical and post-fractured cases. It has been used in 19 studies related to the efficacy of orthotic management and in 31 studies related to the outcomes of surgical corrections [11, 25]. Furthermore, it has been employed in numerous cross-sectional and observational studies.

Some limitations are highlighted in the current study; for example, the sample participants were young and the recruitment was done at a single tertiary care hospital.

## Conclusions

The FFI-Ar proved to be a feasible, valid and reliable outcome measure for assessing both traumatic and non-traumatic foot and ankle disorders, for Arabic-speaking patients.

## Supplementary Information

**Additional file 1.** **Appendix.** Translation of FFI into different languages.

## Data Availability

The datasets used and/or analysed during the current study are available from the corresponding author on reasonable request.

## Abbreviations

FFI: Foot Function Index
FFI-Ar: Foot Function Index for Arabic-speaking patients
PROMs: Patient-reported outcome measures
ISPOR: International Society for Pharmacoeconomics and Outcomes Research
NRS: Numeric rating scale
GRC: Global rating of change scale
SF-36: Short Form 36 health survey
PF: Physical functioning
RP: Role limitation due to physical health problems
BP: Bodily pain
GH: General health perceptions
VT: Vitality, energy and fatigue
SF: Social functioning
RE: Role limitation due to emotional problems
SPSS: Statistical Package for the Social Sciences
ICC: Intraclass correlation coefficient
