# Supplementary material for: Foot Function Index for Arabic-speaking patients (FFI-Ar): translation, cross-cultural adaptation and validation study
Source: J Orthop Surg Res. 2022 Apr 7;17:212. doi: 10.1186/s13018-022-03092-7 (PMC8991848; doi:10.1186/s13018-022-03092-7)
Supplement: Supplementary file 1 — Additional file 1. Appendix. Translation of FFI into different languages. [file 13018_2022_3092_MOESM1_ESM.docx]

**Appendix**

**Translation of FFI into different languages**

| S.No | Translated Version | Year | Sample size (n) | Psychometric properties | Other instrument for Criterion validity | Pathologies for inclusion criteria | Conclusion |
| --- | --- | --- | --- | --- | --- | --- | --- |
| 1 | Thai version of FFI (FFI-Th) (1) | 2020 | 49 | Internal Consistency  Total score (CA = 0.974)  Pain (CA = 0.946)  Disability (CA= 975)  Activity Limitation (CA =714) | - VAS-Pain - EuroQol five-dimension questionnaire   (EQ-5D-5L) | Pain or tenderness at the plantar surface of the foot during rest and/or during prolonged weight-bearing activities of at least 1 month | The FFI-Th was a reliable and valid questionnaire to assess the foot function in a Thai population |
| 2 | Persian version of FFI  (FFI-Per) (2) | 2019 | 113 | Internal Consistency  Pain (CA = 0.90)  Disability (CA = 0.93)  Activity limitation (CA = 0.88)  Total (CA = 0.95) | - Manchester Oxford foot questionnaire (MOXFQ) | Polydactyly, Brachymetatarsia, Sinus Tarsi, Hallux Valgus, Hallux Rigidus, ankle sprain, Plantar Fasciitis, Metatarsal Giant Tumor, Talar Deformity, Freiburg Deformity, Haglund Deformity, Talar OCD, Charcot Foot, Calcaneal Cyst, Os Naviculare, | FFI is valid and reproducible in Persian speaking population. |
| 3 | Turkish version of FFI  (FFI-Tr) (3) | 2018 | 159 | Cronbach’s alpha ranged between 0.821 and 0.938.  Intraclass correlation coefficient values ranged between 0.960 and 0.985 | - SF-36 | Plantar fasciitis, hallux valgus, pes planus, and hammertoe deformities. | FFI-TR is a reliable and valid scale and can be used in Turkish speaking population. |
| 4 | Korean version of FFI  (FFI-Kor)(4) | 2017 | 36 | Test re-test reliability  Pain: 0.9  Disability: 0.94  Activity limitation: 0.91 | - SF-36 - VAS | Plantar fasciitis and foot/ankle fracture. | Korean version is a reliable and valid instrument for assessing foot complaints. |
| 5 | Brazilian Portuguese version of FFI  (FFI-BP) (5) | 2016 | 50 | ICC range of 0.99–0.97  Cronbach’s alpha range of 0.80–0.61 | - SF-36 - Foot and Ankle Outcome Score   (FAOS) | Plantar fasciitis, metatarsalgia and  chronic ankle sprain. | The Brazilian‑Portuguese version of the FFI questionnaire was found to be a valid and reliable instrument for foot function evaluation. |
| 6 | Danish version of FFI (FFI-DK) (6) | 2015 | 35 | Internal Consistency  Pain: 0.99  Disability:0.98  Activity limitation: 0.98  Total: 0.97 | No PROM used | Foot and ankle complaints | Danish version of FFI was found to be valid and reliable and therefore acceptable for use in the Danish population. |
| 7 | French validation of FFI (FFI-Fr) (7) | 2015 | 53 | Cronbach’s alpha ranged from 0.85 to 0.97  correlation coefficients > 0.56 | - VAS-Pain - Health Assessment Questionnaire - McMaster Toronto Arthritis questionnaire | Rheumatoid arthritis | This tool can be used in routine practice and clinical research for evaluating the rheumatoid foot and other pathologies with foot-related functional impairments. |
| 8 | Spanish version of FFI (FFI-Sp) (8) | 2014 | 80 | Internal Consistency  pain 0.95; disability 0.96; and activity limitation 0.69 | - EuroQol 5-D      - VAS Pain - Short Form SF-12 Health Survey | Plantar fasciitis  Osteoarthritis  Nail pathology | The Spanish version of the Foot Function Index (FFI-Sp) is a tool that is a valid and reliable tool with a very good internal consistency for use in the assessment of pain, disability and limitation of the function of the foot, for use both in clinic and research |
| 9 | Taiwan Chinese version of FFI (9) | 2008 | 88 | Cronbach’s α=0.94  ICC = 0.82 | Short form 36  (SF-36) | Plantar fasciitis  Foot and ankle fracture | The adapted Taiwan Chinese version of the FFI is reliable and valid and can be applied among traumatic and non-traumatic foot disorders. |
| 10 | Chinese cultural adaptation of FFI (10) | 2018 | 306 | Internal consistency ranged from 0.996 to 0.998  Test–retest analysis ranged from 0.985 to 0.994 | - FFI (Taiwan Chinese version) - SF-12 Chinese version - EuroQol-5D Chinese version | Neuromusculoskeletal disease in foot/ankle | Foot Function Index Chinese version psychometric characteristics were good to excellent |
| 11 | German  Translation of FFI (FFI-D)  (11) | 2008 | 53 | Internal consistency: 0.97  Test-retest reliability 0.98 | - Short Form SF-36 Health Survey - VAS pain - VAS function - University of California at Los Angeles (UCLA) activity scale | Foot surgery | The German  version of the FFI is a reliable and valid questionnaire for  the self-assessment of pain and disability in German-speaking  patients with foot complaints. |
| 12 | Italian translation of FFI (FFI-It)  (12) | 2013 | 89 | Internal consistency: 0.95  Test-retest reliability ICC Pain: 0.94  ICC Disability: 0.91 | - Short Form (SF-36) Health Survey - Visual Analogue Scale (VAS) | Hallux valgus, Hallux rigidus), Lisfranc injuries, ankle/ subtalar osteoarthritis, osteochondral lesion of the talus | The Italian version of the FFI showed sat- isfactory psychometric properties in Italian patients with foot and ankle diseases. Further testing in different and larger samples is required in order to ensure the validity and reliability of this score. |

**References:**

1. Bovonsunthonchai S, Thong-On S, Vachalathiti R, Intiravoranont W, Suwannarat S, Smith R. Thai version of the foot function index: A cross-cultural adaptation with reliability and validity evaluation. BMC Sports Sci Med Rehabil. 2020;12(1):1–11.

2. Mousavian A, Mohammadi A, Hosseinian S, Shahpari O. Reliability and Validity of the Persian Version of the Foot Function Index in Patients with Foot Disorders. Arch Bone Jt Surg. 2019;291(5):291–6.

3. Külünkoğlu BA, Firat N, Yildiz NT, Alkan A. Reliability and validity of the Turkish version of the Foot Function Index in patients with foot disorders. Turkish J Med Sci. 2018;48:476–83.

4. In T, Jung J, Kim K. The reliability and validity of the Korean version of the foot function index for patients with foot complaints. J Phys Ther Sci Orig. 2017;29:53–6.

5. Martinez BR, Staboli IM, Kamonseki DH, Budiman-Mak E, Yi LC. Validity and reliability of the Foot Function Index (FFI) questionnaire Brazilian-Portuguese version. Springerplus. 2016;5(1).

6. Jorgensen JE, Andreasen J, Rathleff MS. Translation and validation of the Danish Foot Function Index (FFI-DK). Scand J Med Sci Sport. 2015;25(4):e408–13.

7. Pourtier-Piotte C, Pereira B, Soubrier M, Thomas E, Gerbaud L, Coudeyre E. French validation of the Foot Function Index (FFI). Ann Phys Rehabil Med [Internet]. 2015;58(5):276–82. Available from: http://dx.doi.org/10.1016/j.rehab.2015.07.003

8. Paez-Moguer J, Budiman-Mak E, Cuesta-Vargas AI. Cross-cultural adaptation and validation of the Foot Function Index to Spanish. Foot Ankle Surg [Internet]. 2014;20(1):34–9. Available from: http://dx.doi.org/10.1016/j.fas.2013.09.005

9. Wu S, Liang H, Hou W. Reliability and Validity of the Taiwan Chinese Version of the Foot Function Index. J Formos Med Assoc. 2008;107(2):111–22.

10. González-Sánchez M, Ruiz-Muñoz M, Li GZ, Cuesta-Vargas AI. Chinese cross-cultural adaptation and validation of the Foot Function Index as tool to measure patients with foot and ankle functional limitations. Disabil Rehabil [Internet]. 2018;40(17):2056–61. Available from: https://doi.org/10.1080/09638288.2017.1325944

11. Naal FD, Impellizzeri FM, Huber M, Rippstein PF. Cross-Cultural Adaptation and Validation of the Foot Function Index for Use in German-Speaking Patients with Foot Complaints. Foot Ankle Int. 2008;29(12):1222–8.

12. Gennaro M, Scotto M, Bianchi A, Malerba F. Reliability , validity and responsiveness of the Italian version of the Foot Function Index in patients with foot and ankle diseases. Qual Life Res. 2014;23:277–84.
